# A minimally invasive multiple marker approach allows highly efficient detection of meningioma tumors

**DOI:** 10.1186/1471-2105-7-539

**Published:** 2006-12-21

**Authors:** Andreas Keller, Nicole Ludwig, Nicole Comtesse, Andreas Hildebrandt, Eckart Meese, Hans-Peter Lenhof

**Affiliations:** 1 Center for Bioinformatics, Saarland University, Building 3.11, 66041 Saarbrücken, Germany; 2 Department of Human Genetics, Medical School, Saarland University, Building 60, 66421 Homburg/Saar, Germany

## Abstract

**Background:**

The development of effective frameworks that permit an accurate diagnosis of tumors, especially in their early stages, remains a grand challenge in the field of bioinformatics. Our approach uses statistical learning techniques applied to multiple antigen tumor antigen markers utilizing the immune system as a very sensitive marker of molecular pathological processes. For validation purposes we choose the intracranial meningioma tumors as model system since they occur very frequently, are mostly benign, and are genetically stable.

**Results:**

A total of 183 blood samples from 93 meningioma patients (WHO stages I-III) and 90 healthy controls were screened for seroreactivity with a set of 57 meningioma-associated antigens. We tested several established statistical learning methods on the resulting reactivity patterns using 10-fold cross validation. The best performance was achieved by Naïve Bayes Classifiers. With this classification method, our framework, called Minimally Invasive Multiple Marker (MIMM) approach, yielded a specificity of 96.2%, a sensitivity of 84.5%, and an accuracy of 90.3%, the respective area under the ROC curve was 0.957. Detailed analysis revealed that prediction performs particularly well on low-grade (WHO I) tumors, consistent with our goal of early stage tumor detection. For these tumors the best classification result with a specificity of 97.5%, a sensitivity of 91.3%, an accuracy of 95.6%, and an area under the ROC curve of 0.971 was achieved using a set of 12 antigen markers only. This antigen set was detected by a subset selection method based on Mutual Information. Remarkably, our study proves that the inclusion of non-specific antigens, detected not only in tumor but also in normal sera, increases the performance significantly, since non-specific antigens contribute additional diagnostic information.

**Conclusion:**

Our approach offers the possibility to screen members of risk groups as a matter of routine such that tumors hopefully can be diagnosed immediately after their genesis. The early detection will finally result in a higher cure- and lower morbidity-rate.

## Background

Tumor markers have been established to detect cancer, to monitor cancer progression, to gauge responsiveness to cancer treatment, and to provide insight into tumor development. Molecular tumor markers can be grouped into those that are identifiable in cancer cells and those that are secreted as molecules into body fluids. Markers of the first group encompass a wide spectrum including chromosome alterations, epigenetic DNA modifications, altered RNA and protein expression, and protein modifications. Detection of these markers requires the availability of cancer cells either obtained by tumor biopsies or by cancer cell isolation from blood or other body fluids. The isolation of cancer cells from body fluids and their use as markers is still in its early stages. The requirement of a tumor biopsy limits the usefulness of such markers for early detection of cancer. Among the second group of markers the prostate specific antigen (PSA) is one of the few markers that are widely used in diagnosis and monitoring of cancer [1].

However, even PSA has its severe limitations both in detection and monitoring of prostate cancer. PSA is found at high levels in approximately one third of the patients without prostate cancer and its benefits for monitoring after treatment remain controversial. Other serum markers like CA-15.3 for breast cancer and CA-19.9 for pancreatic cancer also have severe limitations [2]. Mass spectroscopy is an up-to-date method to perform minimally invasive cancer detection. A promising approach using Matrix-Assisted Laser Desorption and Ionization (MALDI) mass spectroscopy evaluated by 'peak probability contrasts' revealed an accuracy of around 70% for ovarian cancer [3]. Similar approaches for pancreatic cancer performed slightly better with 88% sensitivity and 75% specificity [4].

The onset of autoantibody signatures paved the way not only for an improved diagnosis but also for a new kind of monitoring of molecular processes in early tumor development. Wang and co-workers [5] reported an autoantibody signature that allows for detection of prostate cancer with 88.2% specificity and 81.6% sensitivity. However, a limitation of the study was the use of many peptides with weak homology to known proteins termed mimotopes. Most recently, a study of ovarian cancer based on Bayesian modeling showed similarly good results [6]. Prior to the work by Erkanli and co-workers, we reported a first study that identified a complex antibody response in patients with meningioma [7]. Here, we present a novel concept for a serum-based diagnosis of human tumors, especially in their early stages of development. We chose meningioma as a model, which is a priori not expected to trigger a complex immune response: first meningioma is a generally benign tumor, and second it is genetically rather stable. Both factors do not favor a complex immune response. Our approach permits the separation of meningioma sera and normal sera with high specificity and sensitivity, especially the separation of low-grade common type meningiomas (WHO I) and normal controls. To reach this high performance, we screened a total of 183 blood samples from 93 meningioma patients (WHO stages I-III) and 90 individuals without known disease (controls) for seroreactivity with a set of 57 meningioma-associated antigens, i.e. antigens that were previously found in sera of meningioma patients. Having screened the 183 sera for these antigens we can group the meningioma-associated antigens in two subgroups. Antigens that are found in at least one of the 93 meningioma sera but not in any of the 90 control sera are denoted as meningioma-specific antigens. All antigens that are detected in at least one of the 93 meningioma sera but also in at least one of the 90 control sera are denoted as non-specific antigens. We show in our study that the identification of meningiomas, especially of low-grade common type meningiomas, can be carried out with a significantly decreased subset of antigens that includes meningioma-specific as well as non-specific antigens.

## Results

### Mutual information of specific and non-specific antigens

**Figure 1:**
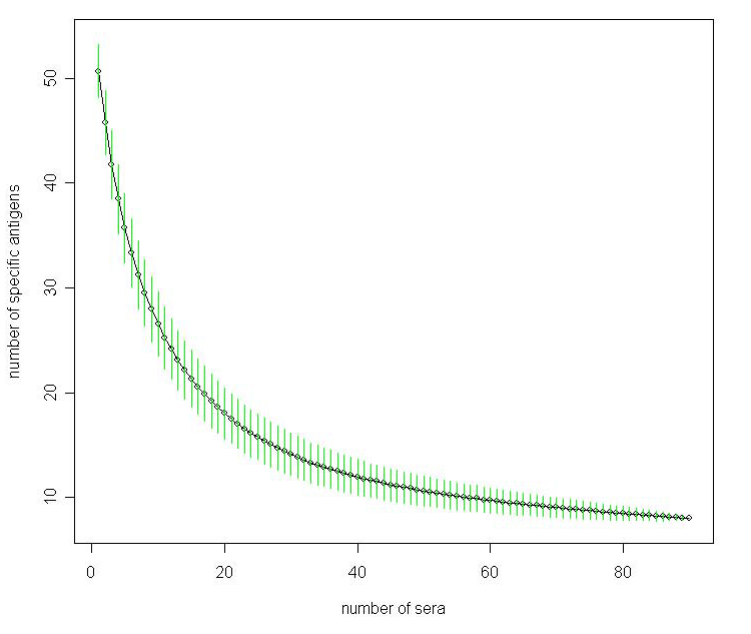
**Decrease of specific antigens**. Decrease of meningioma-specific antigens in the samples as a function of the number of screened normal sera computed by random sampling. Standard deviations of each subset size are shown as vertical green bars.

One of the original goals of our project was to define a set of meningioma-specific antigens that react with meningioma sera but not with normal sera. With increasing number of normal sera we found a decreasing number of meningioma-specific antigens as indicated in Figure 1.

**Figure 2:**
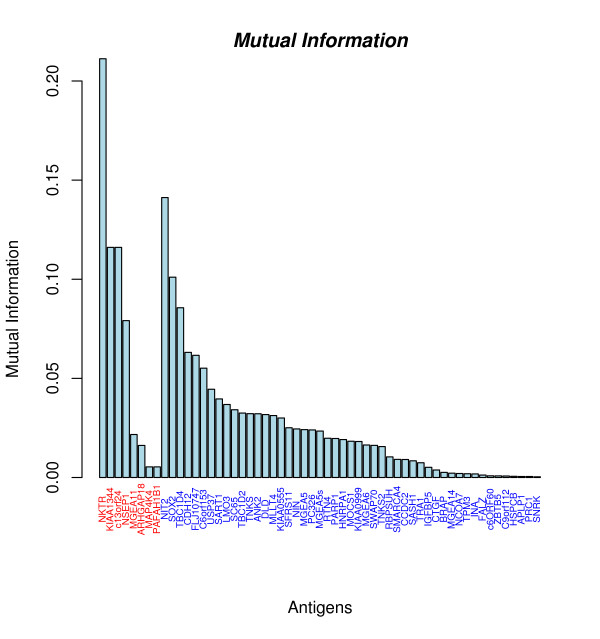
**Mutual Information of 57 antigens**. Meningioma specific antigens are colored red. Notably, the antigen with the second highest mutual information (NIT2) reacts with meningioma and normal sera.

Notably, 49 of 57 antigens (86%) are detected in meningioma sera and normal sera. One reason for the occurrence of non-specific antigens that can not be ruled out completely is false positive antigen reactivity that is of course unavoidable, especially when large numbers of sera are analyzed. Any false positive antigen reactivity in a normal serum possibly converts a specific antigen into a non-specific antigen. However, our study shows that some non-specific antigens entail even more information for the diagnostic task than most of the specific antigens. The mutual information as explained in 'Methods' offers an appropriate measure of the information content of an antigen. The mutual information values of all antigens are shown in Figure 2. For meningioma-specific antigens the mutual information ranges between 0.005 and 0.211 with a mean value of 0.071 and a median of 0.05, whereas for non-specific antigens it ranges between 0 and 0.141 with a mean of 0.024 and a median of 0.018. As detailed in Figure 2 and Table 1, many non-specific antigens provide even more mutual information than the majority of specific antigens. An example of such an antigen is NIT2 with a mutual information value of 0.141. Notably, such a high value is reached by only 1 of the 8 specific antigens. Even more, the difference of the mutual information of specific and non-specific antigens was statistically not significant (p-value of 0.09, unpaired two sample Wilcoxon Mann-Whitney test). These findings support our hypothesis, that non-specific antigens are suitable to enhance meningioma detection.

**Table 1:** Information about antigens and antigen reactivity

Gene	Meningioma reac. (%)	Normal reac. (%)	Quotient	Mutual Information	Known Protein
NKTR	0,37	0,00	na	0,21	yes
NIT2	0,37	0,03	10,97	0,14	yes
KIAA1344	0,22	0,00	na	0,12	no
c13orf24	0,22	0,00	na	0,12	yes
SOX2	0,24	0,01	21,29	0,10	yes
TBC1D4	0,29	0,04	6,53	0,09	yes
NSEP1	0,15	0,00	na	0,08	yes
CDH12	0,32	0,09	3,63	0,06	yes
FLJ10747	0,30	0,08	3,87	0,06	yes
C6orf153	0,15	0,01	13,55	0,06	no
USP37	0,13	0,01	11,61	0,04	yes
SART1	0,39	0,18	2,18	0,04	yes
LMO3	0,14	0,02	6,29	0,04	yes
SC65	0,22	0,07	3,23	0,03	yes
TBC1D2	0,42	0,22	1,89	0,03	yes
TNKS	0,13	0,02	5,81	0,03	yes
ANK2	0,17	0,04	3,87	0,03	yes
DLD	0,15	0,03	4,52	0,03	yes
MLLT4	0,04	0,17	0,26	0,03	yes
KIAA0555	0,40	0,21	1,88	0,03	yes
SFRS11	0,27	0,12	2,20	0,03	yes
NIN	0,20	0,08	2,63	0,02	yes
MGEA5	0,15	0,04	3,39	0,02	yes
PC326	0,32	0,17	1,94	0,02	yes
MGEA5s	0,13	0,03	3,87	0,02	yes
MGEA11	0,04	0,00	na	0,02	yes
RTN4	0,16	0,30	0,54	0,02	yes
PARP1	0,33	0,19	1,76	0,02	yes
HNRPA1	0,41	0,26	1,60	0,02	yes
MOCS1	0,22	0,10	2,15	0,02	yes
KIAA0999	0,15	0,06	2,71	0,02	yes
MGEA6	0,16	0,07	2,42	0,02	yes
SWAP70	0,24	0,12	1,94	0,02	yes
ARHGAP18	0,03	0,00	na	0,02	yes
TNKS2	0,60	0,46	1,32	0,02	yes
RBPSUH	0,23	0,33	0,68	0,01	yes
SMARCA4	0,02	0,07	0,32	0,01	yes
CCDC2	0,12	0,06	2,13	0,01	yes
SASH1	0,22	0,13	1,61	0,01	yes
TRA1	0,18	0,11	1,65	0,01	yes
MAP4K4	0,01	0,00	na	0,01	yes
PAFAH1B1	0,01	0,00	na	0,01	yes
IGFBP5	0,13	0,08	1,66	0,01	yes
CTGF	0,11	0,07	1,61	0,00	yes
BRAP	0,54	0,48	1,13	0,00	yes
MGEA14	0,10	0,07	1,45	0,00	yes
NCOA7	0,13	0,17	0,77	0,00	yes
TPM3	0,11	0,08	1,38	0,00	yes
INA	0,19	0,16	1,24	0,00	yes
FALZ	0,02	0,01	1,94	0,00	yes
c6ORF60	0,27	0,30	0,90	0,00	no
ZBTB5	0,10	0,08	1,24	0,00	yes
C9orf112	0,22	0,19	1,14	0,00	no
HSPCB	0,12	0,10	1,18	0,00	yes
APLP1	0,05	0,07	0,81	0,00	yes
PRC1	0,28	0,26	1,09	0,00	yes
SNRK	0,27	0,29	0,93	0,00	yes

### Classification of sera using all antigens

**Figure 3:**
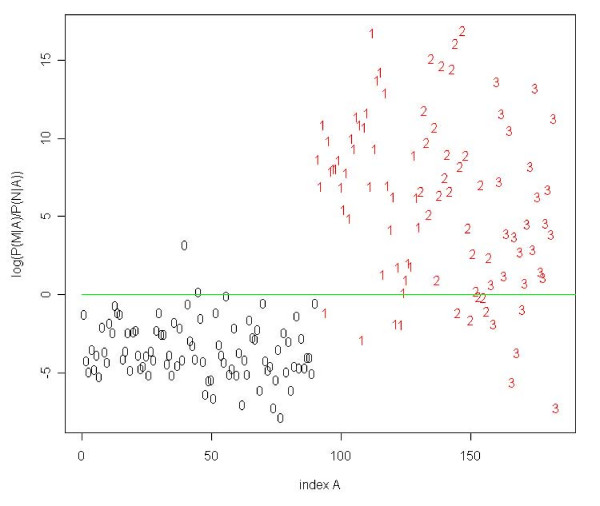
**Classification results**. Logarithm of the quotient *Q*(*A*) of *P*(*M*|*A*) over *P*(*N*|*A*) for each of the 183 sera. Normal sera are colored black, meningioma sera red. Numbers denote the corresponding WHO grade of each serum. Using a threshold of 1 (green line), we classify two normal sera, four sera of patients with a common type meningioma, and five sera of patients with a WHO grade II and grade III meningioma not correct.

We applied several standard classification methods to the complete set of 93 meningioma and 90 normal sera that were evaluated by using 10-fold cross validation. The first Naïve Bayes approach, introduced in the 'Materials' section, reached a specificity of 96.2% (95%-CI = [96.0%, 96.5%]), a sensitivity of 84.5% (95%-CI = [84.3%, 84.8%]), an accuracy of 90.3% (95%-CI = [90.1%, 90.4%]), and an AUC (area under the curve) value of 0.957 (95%-CI = [0.956, 0.957]). The classification result of an arbitrary selected cross-validation run is shown in Figure 3. The second Bayes approach showed similar performance with a slightly increased specificity of 97.0% (95%-CI = [96.8%, 97.1%]), a sensitivity of 83.8% (95%-CI = [83.5%, 84.0%]), and an accuracy of 90.3% (95%-CI = [90.1%, 90.4%]). We tested the data with several other statistical learning methods (among them for example Support Vector Machine, Linear Discriminant Analysis) that yielded similar high-quality classification results, indicating the high information content of the antigen profiles. In order to validate our approaches, we carried out 100 permutation tests by randomly permuting class labels before classifying the 183 sera. The randomly permuted data yielded an averaged accuracy of 50%, which corresponds to random guessing. The best random test showed an accuracy of only 70%. An unpaired two-sample Wilcoxon Mann-Whitney test yielded a p-value of smaller 10^-10^, asserting that the above classification results can be attributed to the information content of the data set and not to chance.

### Classification of common type meningioma using all antigens

Since we are especially interested to perform accurate diagnoses of early stages of tumor development we classified low-grade common type meningioma (WHO grade I) sera versus normal sera. Using the complete set of 57 antigens, common type meningioma sera are separated from normal sera with a specificity of 98.6% (95%-CI = [98.5%, 98.8%]), a sensitivity of 87.5% (95%-CI = [87.3%, 87.6%]), and an accuracy of 95.2% (95%-CI = [95.0%, 95.3%]). The respective AUC value was 0.967 (95%-CI = [0.966, 0.967]). For comparison, we also classified the sera of grade II and III tumor patients. The results of the classification are summarized in Table 2. The classification result of the first Naïve Bayes approach shown in Figure 3 indicates that sera of common type and atypical meningiomas can be clearly differentiated from sera of healthy individuals whereas WHO grade III sera cannot be equally well separated from normal sera. This finding is reflected in the high AUC values for the detection of WHO I and II sera and the relatively small AUC value for the detection of WHOIII sera. Applying unpaired two-sample t-tests, we found that the differences of the classification results were statistically significant in each case (p-value < 0.0002).

**Table 2:** Classification results

	Spec.	Sens.	Acc.	AUC
Meningioma (93)/Normal (90)	96.2%	84.5%	90.3%	0.957
WHO I(40)/Normal (90)	98.6%	87.5%	95.2%	0.967
WHO II (27)/Normal (90)	98.8%	67.6%	91.8%	0.969
WHO III (26)/Normal (90)	97.9%	72.2%	92.1%	0.901

### Number of antigens required for classification

**Figure 4:**
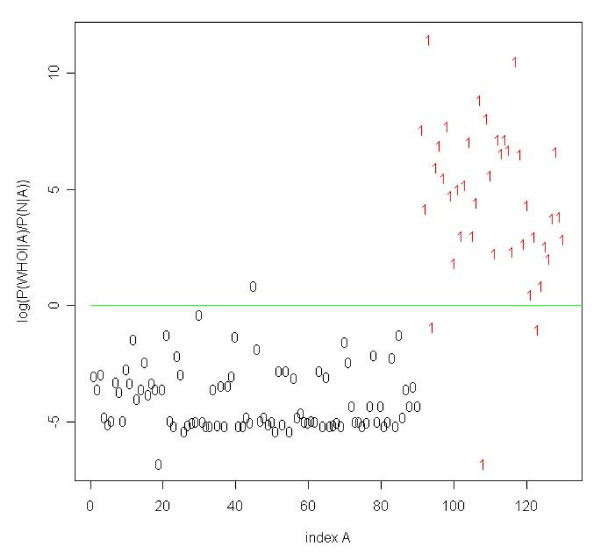
**Classification results of WHO grade I meningioma**. Classification results of low-grade (WHO grade I) meningiomas using shrunken antigen subset. Using 12 antigens only, we classify one normal serum and three WHO grade I sera not correct.

**Figure 5:**
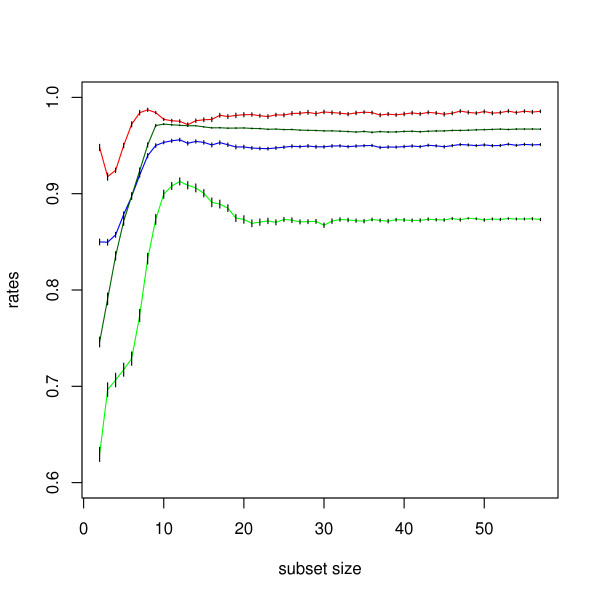
**Result of the subset selection**. Specificity (red), sensitivity (green), accuracy (blue), and area under the ROC curve (black) as a function of the number of antigens used to separate common type meningioma sera from normal sera. With a subset size of only 12 antigens the Naïve Bayes approach reaches a specificity of 97.5%, a sensitivity of 91.3%, and an accuracy of 95.6%. The respective AUC value is 0.971. The 95% confidence intervals are indicated as vertical black bars.

Next, we computed the minimal number of antigens required for an optimal classification of low-grade meningiomas using the subset selection method described in 'Materials'. Twelve antigens only yielded the best separation between common type meningiomas and normal sera with a specificity of 97.5% (95%-CI = [97.4%, 97.7%]), a sensitivity of 91.3% (95%-CI = [90.9%, 91.6%]), and an accuracy of 95.6% (95%-CI = [95.4%, 95.8%]). The corresponding AUC value was 0.971 (95%-CI = [0.970, 0.971]). Notably, not all of these 12 antigens are meningioma-specific. One classification result is shown exemplarily in Figure 4. The specificity, sensitivity, accuracy, and AUC value as a function of the number of antigens are provided in Figure 5. This result indicates that the identification of low-grade meningiomas with high specificity and sensitivity requires only a subset of all antigens. For comparison, we also carried out the same subset selection procedure for WHO grade II and III sera. An optimal classification of WHO grade II meningioma sera from normal sera requires 36 antigens resulting in a specificity of 98.9% (95%-CI = [98.8%, 98.9%]), a sensitivity of 70.1% (95%-CI = [69.6%, 70.6%]), an accuracy of 92.2% (95%-CI = [92.1%, 92.3%]), and an AUC of 0.969 (95%-CI = [0.968, 0.971]). For WHO grade III, 53 antigens are necessary to perform an optimal classification, yielding a specificity of 97.9% (95%-CI = [97.7%, 98.0%]), a sensitivity of 73.9% (95%-CI = [73.2%, 74.7%]), an accuracy of 92.5% (95%-CI = [92.2%, 92.7%]), and an AUC of 0.902 (95%-CI = [0.900, 0.904]). The classification results are summarized in Table 3.

**Table 3:** Classification results using feature subset selection

	Subset Size	Spec.	Sens.	Acc.	AUC
Meningioma/Normal	38	96.4%	85.1%	90.6%	0.959
WHO I/Normal	12	97.5%	91.3%	95.6%	0.971
WHO II/Normal	36	98.9%	70.1%	92.2%	0.969
WHO III/Normal	53	97.9%	73.9%	92.5%	0.902

Performing the classification of normal sera versus meningioma sera by using just the 8 specific antigens reduced the accuracy and AUC value significantly to 80% and 0.78 (p-value < 10^-10^, unpaired two sample Wilcoxon Mann-Whitney test). Therefore, integration of the non-specific antigens that contribute additional information makes the classification significantly more accurate and reliable.

## Discussion

The availability of a set of immunogenic antigens is central to the idea of using the reactivity pattern to gain insight into the molecular pathology of tumor development. Many antigens formerly considered as tumor specific antigens also show reactivity with normal sera if the number of screened normal sera is increased. Scanlan and co-workers propose that approximately 60% of cancer antigens react with normal sera [8]. Likewise, the definition of tumor antigens based on the expression pattern is less clear than originally proposed. A ubiquitous expression is reported for more than 10% of cancer testis antigens that should by definition be expressed in testis and cancer only [9]. Our results are consistent with this data in that they also show a decreasing number of specific antigens with increasing number of normal sera. A lack of tumor-specific antigens, i.e. antigens that do not react with normal sera, is generally thought to impair the development of antigens sets useful for tumor analysis. However, our study shows for the first time that the observed decrease of the number of specific antigens with increasing number of screened normal sera poses no problem. In fact, including non-specific antigens in the marker set improved the accuracy and reliability of the serum based approach significantly.

We have shown that our diagnosis works especially well on low-grade (WHO grades I and II) meningioma sera. That observation can be explained by the fact that the sera of lower-grade meningiomas show on average an increased immune response compared to WHO grade III sera. On averaged, 11.8 of the 57 antigens show reactivity with WHO grade I sera, 12.1 with WHO grade II sera, and 10.8 with WHO grade II sera. In comparison, normal sera show an averaged reactivity of only 6.3 antigens per serum. The decrease of seroreactivity in WHO grade III tumors may be a result of antigen loss as part of a tumor escape mechanism [10].

The knowledge of the nature of the antigens is of great value for a serum-based analysis of human tumors. A recent study shows a relatively high number of sequences that do not represent known proteins [5]. These sequences are thought to mimic immunogenic antigens (mimotopes). Without having high homology to known proteins, mimotopes are of no use to provide insight into tumor development. In our study, 53 of the 57 marker sequences (93%) are homologous to known proteins as shown in Table 1.

To further evaluate our set of meningioma-associated antigens, we computed the overlap of the 57 meningioma-associated antigens with the antigen sets that were reported for ovarian and prostate cancer types [11,5]. We found no overlap with any of these antigen sets. An analysis using PubMed showed that only six meningioma-associated antigens (10.5%) were immunogenic in other human cancers. These results indicate that our set of meningioma-associated antigens very likely classifies only sera of meningioma patients as meningioma sera.

As addressed above, experimental approaches always bear the possibility of misclassifications. In our study, we misclassified a small number of normal sera as meningioma sera (false positive predictions). This leaves the question whether a positive prediction of a normal serum is a classification error or represents a not detected meningioma patient. According to our protocol, all normal sera were randomized prior to the experiments. This protocol excluded the possibility to examine donors of control blood sera for a potential tumor. It cannot be ruled out that our test identified a tumor patient that has so far gone unnoticed. While the annual incidence of meningioma patients that come to attention in the clinic is approximately 6 in 10^5 ^[12], post mortem studies suggest a true incidental asymptomatic rate of approximately 1.4% [12]. The comparatively high prevalence results in an excellent negative predictive value of 0.99 and an acceptable positive predictive value of 0.56.

MIMM neither depends on a single marker nor represents a proteomics approach like the serum based diagnosis of ovarian cancer that triggered a discussion over the general validity of any diagnostic test based on proteomics [13]. Unlike many proteomics approaches, MIMM utilizes a small set of proteins only. It is a conservative approach in that any additional serum analysis helps to improve the set of antigens that best marks out a cancer patient. MIMM is an open system that is designed to constantly improve over time. Once a critical group of antigens is assembled for a given cancer type, any investigator can add, or if necessary remove, antigens to optimize the power of an antigen set for the characterization of patients with a specific tumor. In addition, any new serum that is analyzed with the antigen set improves the predictive value of each antigen of the set. These results indicate that our approach appears to be well suited to analyse the majority of meningioma patients and to do so specifically efficient for patients with low-grade meningiomas. Provided these results can be extended to other tumor types, MIMM represents a highly promising approach to analyse tumors that are still in its early stages of development.

## Conclusion

We presented a minimally invasive diagnostic framework based on the classification of tumor antigen patterns in blood sera using statistical learning techniques. We validated our approach on meningioma tumors finding that it is especially suited to detect tumors that are still in their early stages of development. To further validate and improve the presented approach, independent training and test set of appropriate size will be generated. Since our long term goal is a diagnostic framework for a broad range of human tumors we will test MIMM on several other tumor types. Our diagnostic tool may offer the possibility to screen members of risk groups at regular intervals such that tumors can be diagnosed immediately after their genesis. It can be expected that the early detection will finally result in a higher cure- and lower morbidity-rate [14].

## Methods

### Sera and antigens

By screening a fetal brain expression library with meningioma patients' sera, we previously identified 57 meningioma-associated antigens [7]. Information about the antigens is provided in Table 1. To establish an analysis tool to distinguish meningioma patients' sera from control sera of healthy persons, we used this set of antigens to screen 93 patients' sera (40 WHO grade I, 27 WHO grade II, and 26 WHO grade III) and 90 healthy controls with the spot assay method. Informed consent was obtained from patients for use of blood sera. The age of the 93 patients ranged between 31 to 85 years, with a mean value of 60.5 years and a standard deviation of 11.7 years. Out of 93 patients, 64 were females and 29 were males. All normal sera were randomly selected. Blood serum was taken from meningioma patients directly before surgery. For serum preparation, blood was collected in 10-ml serum gel monovettes and centrifuged for 10 min. The serum was stored as 2 ml aliquots at -70°C.

### Antigen screening

Standard SEREX was used to isolate antigens from a fetal brain expression library using sera from meningioma patients. The antigen set was screened by serological spot assay as described in [7]. In brief, E. coli XL1 blue MRF were transfected with recombinant lambda phages and spotted onto nitrocellulose membranes that were precoated with a layer of NZCYM/0.7% agarose/0.25 M IPTG. After overnight incubation the agarose layer was removed and membranes were processed for reactivity with individual sera samples at a 1:100 dilution. The seroreactivity patterns of all sera are freely available upon request.

### Classification methods

The screening of the 93 meningioma sera and 90 normal sera yielded a 183 × 57 binary matrix containing a '1' at position (*i, j*) if antigen *j *has been detected in serum *i *and a '0' otherwise. The antigen pattern *A *of serum *i *is represented by the *i*-th row of the matrix. In order to identify a suitable classification algorithm, we tested several standard statistical learning methods like Support Vector Machines (SVM) or Linear Discriminant Analysis (LDA) (For a survey of these techniques we refer to [15]. The best results were obtained with two different Naïve Bayes Classifiers. The first Bayes approach computes the probabilities *P*(*M*|*A*) and *P*(*N*|*A*) of a given antigen pattern *A *representing a meningioma or a normal serum. If the quotient of *P*(*M*|*A*) over *P*(*N*|*A*) is larger than a chosen threshold *t*, the serum is classified as meningioma serum and otherwise as normal serum. A sensible choice for the threshold parameter *t *is 1. Increasing the threshold results in a higher specificity and decreasing the threshold leads to a higher sensitivity. The second Bayes approach computes the four conditional probabilities *P*(*N*|*A*), *P*(*MI*|*A*), *P*(*MII*|*A*), and *P*(*MIII*|*A*), where the latter three probabilities represent the three meningioma grades. These three classes are unified to one 'meningioma class', i.e., if one of the conditional probabilities *P*(*MI*|*A*), *P*(*MII*|*A*), or *P*(*MIII*|*A*) is greater or equals *P*(*N*|*A*), the serum with antigen pattern *A *is classified as meningioma serum. The classification methods were evaluated by 10-fold cross validation. Since different cross validation runs provide different results, the presented results are averaged over 100 runs. In detail, 100 different, randomly selected partitions in 10 parts were carried out and for each of these 100 partitions the classification results were computed. For each classification, the mean accuracy, sensitivity, and specificity are provided together with the 95% confidence intervals (CI).

### Subset selection based on mutual information

In order to identify a minimal set of antigens that allows for an optimal classification we applied a subset selection method based on mutual information. The mutual information is a well known measure in information theory and was introduced by Shannon [16]. The mutual information of an antigen *s *represents a measure of the information content that *s *provides for the classification task. More precisely, the mutual information *I*(*X, Y*) between two discrete random variables *X *and *Y *is given by *H*(*X*) - *H*(*X*|*Y*). *H*(*X*) is the so-called Shannon Entropy defined as

H(X)=∑i=1kp(xi)log(p(xi)),
 MathType@MTEF@5@5@+=feaafiart1ev1aaatCvAUfKttLearuWrP9MDH5MBPbIqV92AaeXatLxBI9gBaebbnrfifHhDYfgasaacH8akY=wiFfYdH8Gipec8Eeeu0xXdbba9frFj0=OqFfea0dXdd9vqai=hGuQ8kuc9pgc9s8qqaq=dirpe0xb9q8qiLsFr0=vr0=vr0dc8meaabaqaciaacaGaaeqabaqabeGadaaakeaacqWGibascqGGOaakcqWGybawcqGGPaqkcqGH9aqpdaaeWbqaaiabdchaWjabcIcaOiabdIha4naaBaaaleaacqWGPbqAaeqaaOGaeiykaKccbiGae8hBaWMae83Ba8Mae83zaCMaeiikaGIaemiCaaNaeiikaGIaemiEaG3aaSbaaSqaaiabdMgaPbqabaGccqGGPaqkcqGGPaqkcqGGSaalaSqaaiabdMgaPjabg2da9iabigdaXaqaaiabdUgaRbqdcqGHris5aaaa@4BA2@

where each *x*_*i *_denotes one of *k *possible states of the random variable *X*. The conditional entropy *H*(*X*|*Y*) is defined as

H(X|Y)=∑i=1k∑j=1lp(xi,yj)log(p(xi|yj)),
 MathType@MTEF@5@5@+=feaafiart1ev1aaatCvAUfKttLearuWrP9MDH5MBPbIqV92AaeXatLxBI9gBaebbnrfifHhDYfgasaacH8akY=wiFfYdH8Gipec8Eeeu0xXdbba9frFj0=OqFfea0dXdd9vqai=hGuQ8kuc9pgc9s8qqaq=dirpe0xb9q8qiLsFr0=vr0=vr0dc8meaabaqaciaacaGaaeqabaqabeGadaaakeaacqWGibascqGGOaakcqWGybawcqGG8baFcqWGzbqwcqGGPaqkcqGH9aqpdaaeWbqaamaaqahabaGaemiCaaNaeiikaGIaemiEaG3aaSbaaSqaaiabdMgaPbqabaGccqGGSaalcqWG5bqEdaWgaaWcbaGaemOAaOgabeaakiabcMcaPGqaciab=XgaSjab=9gaVjab=DgaNbWcbaGaemOAaOMaeyypa0JaeGymaedabaGaemiBaWganiabggHiLdGccqGGOaakaSqaaiabdMgaPjabg2da9iabigdaXaqaaiabdUgaRbqdcqGHris5aOGaemiCaaNaeiikaGIaemiEaG3aaSbaaSqaaiabdMgaPbqabaGccqGG8baFcqWG5bqEdaWgaaWcbaGaemOAaOgabeaakiabcMcaPiabcMcaPiabcYcaSaaa@5DE3@

where each *y*_*j *_denotes one of *l *possible states of the random variable *Y*, *p*(*x*_*i*_, *y*_*j*_) denotes the joint probability of *x*_*i *_and *y*_*j*_, and *p*(*x*_*i*_|*y*_*j*_) denotes the conditional probability of *x*_*i *_given *y*_*j*_. Thus, the mutual information *I*(*X, Y*) can be considered as the reduction in uncertainty about *X *due to the knowledge of *Y*. In our case, *X *and *Y *are binary random variables. The two possible states of the random variable *X *are 'normal' (*X *= 0) or 'meningioma' (*X *= 1). If we are computing the mutual information of antigen *s*, the discrete random variable *Y *can take the states 's not detected' (*Y *= 0) or 's detected' (*Y *= 1). The higher the value of the mutual information of antigen *s*, the more 'valuable' *s *is for the classification task.

In order to define the minimal subset of antigens, we tested each possible antigen subset size *z *∈ {1, ..., 57} using 10-fold cross validation. For a given subset size *z*, we computed the mutual information of all 57 antigens in each cross validation run and selected the *z *antigens with the highest mutual information to perform the classification.

### Significance testing using Wilcoxon Mann-Whitney and t-test

The Wilcoxon Mann-Whitney test [17,18] is a standard test for comparing two populations. It is applied to test the null hypothesis that the two tested populations come from the same distribution against the alternative hypothesis that the populations differ with respect to their location only. The nonparametric Wilcoxon Mann-Whitney test corresponds to the two sample t-test, however, it does not require that the two populations are normally distributed. Therefore, the t-test is applied only if the two populations are normally distributed. The 'normality' was tested by the Shapiro-Wilk Normality test [19].

### Evaluation of results

To estimate the performance of MIMM, we computed accuracy

accuracy=#Correct Predictions#All Predictions
 MathType@MTEF@5@5@+=feaafiart1ev1aaatCvAUfKttLearuWrP9MDH5MBPbIqV92AaeXatLxBI9gBaebbnrfifHhDYfgasaacH8akY=wiFfYdH8Gipec8Eeeu0xXdbba9frFj0=OqFfea0dXdd9vqai=hGuQ8kuc9pgc9s8qqaq=dirpe0xb9q8qiLsFr0=vr0=vr0dc8meaabaqaciaacaGaaeqabaqabeGadaaakeaacqWGHbqycqWGJbWycqWGJbWycqWG1bqDcqWGYbGCcqWGHbqycqWGJbWycqWG5bqEcqGH9aqpdaWcaaqaaiabcocaJiabdoeadjabd+gaVjabdkhaYjabdkhaYjabdwgaLjabdogaJjabdsha0jabbccaGGqaciab=bfaqjab=jhaYjab=vgaLjab=rgaKjab=LgaPjab=ngaJjab=rha0jab=LgaPjab=9gaVjab=5gaUjab=nhaZbqaaiabcocaJiabdgeabjabdYgaSjabdYgaSjabbccaGiab=bfaqjab=jhaYjab=vgaLjab=rgaKjab=LgaPjab=ngaJjab=rha0jab=LgaPjab=9gaVjab=5gaUjab=nhaZbaaaaa@6683@

sensitivity

sensitivity=#True Positives#True Positives+#False Negatives
 MathType@MTEF@5@5@+=feaafiart1ev1aaatCvAUfKttLearuWrP9MDH5MBPbIqV92AaeXatLxBI9gBaebbnrfifHhDYfgasaacH8akY=wiFfYdH8Gipec8Eeeu0xXdbba9frFj0=OqFfea0dXdd9vqai=hGuQ8kuc9pgc9s8qqaq=dirpe0xb9q8qiLsFr0=vr0=vr0dc8meaabaqaciaacaGaaeqabaqabeGadaaakeaacqWGZbWCcqWGLbqzcqWGUbGBcqWGZbWCcqWGPbqAcqWG0baDcqWGPbqAcqWG2bGDcqWGPbqAcqWG0baDcqWG5bqEcqGH9aqpdaWcaaqaaiabcocaJiabdsfaujabdkhaYjabdwha1jabdwgaLjabbccaGiabdcfaqjabd+gaVjabdohaZjabdMgaPjabdsha0jabdMgaPjabdAha2jabdwgaLjabdohaZbqaaiabcocaJiabdsfaujabdkhaYjabdwha1jabdwgaLjabbccaGiabdcfaqjabd+gaVjabdohaZjabdMgaPjabdsha0jabdMgaPjabdAha2jabdwgaLjabdohaZjabgUcaRiabcocaJiabdAeagjabdggaHjabdYgaSjabdohaZjabdwgaLjabbccaGiabd6eaojabdwgaLjabdEgaNjabdggaHjabdsha0jabdMgaPjabdAha2jabdwgaLjabdohaZbaaaaa@78FE@

and specificity

specificity=#True Negatives#True Negatives+#False Positives
 MathType@MTEF@5@5@+=feaafiart1ev1aaatCvAUfKttLearuWrP9MDH5MBPbIqV92AaeXatLxBI9gBaebbnrfifHhDYfgasaacH8akY=wiFfYdH8Gipec8Eeeu0xXdbba9frFj0=OqFfea0dXdd9vqai=hGuQ8kuc9pgc9s8qqaq=dirpe0xb9q8qiLsFr0=vr0=vr0dc8meaabaqaciaacaGaaeqabaqabeGadaaakeaacqWGZbWCcqWGWbaCcqWGLbqzcqWGJbWycqWGPbqAcqWGMbGzcqWGPbqAcqWGJbWycqWGPbqAcqWG0baDcqWG5bqEcqGH9aqpdaWcaaqaaiabcocaJiabdsfaujabdkhaYjabdwha1jabdwgaLjabbccaGiabd6eaojabdwgaLjabdEgaNjabdggaHjabdsha0jabdMgaPjabdAha2jabdwgaLjabdohaZbqaaiabcocaJiabdsfaujabdkhaYjabdwha1jabdwgaLjabbccaGiabd6eaojabdwgaLjabdEgaNjabdggaHjabdsha0jabdMgaPjabdAha2jabdwgaLjabdohaZjabgUcaRiabcocaJiabdAeagjabdggaHjabdYgaSjabdohaZjabdwgaLjabbccaGiabdcfaqjabd+gaVjabdohaZjabdMgaPjabdsha0jabdMgaPjabdAha2jabdwgaLjabdohaZbaaaaa@7860@

of the results. We also computed positive predictive values (PPV)

PPV=sensitivity⋅PRsensitivity⋅PR+(1−specificity)⋅(1−PR)
 MathType@MTEF@5@5@+=feaafiart1ev1aaatCvAUfKttLearuWrP9MDH5MBPbIqV92AaeXatLxBI9gBaebbnrfifHhDYfgasaacH8akY=wiFfYdH8Gipec8Eeeu0xXdbba9frFj0=OqFfea0dXdd9vqai=hGuQ8kuc9pgc9s8qqaq=dirpe0xb9q8qiLsFr0=vr0=vr0dc8meaabaqaciaacaGaaeqabaqabeGadaaakeaacqWGqbaucqWGqbaucqWGwbGvcqGH9aqpdaWcaaqaaiabdohaZjabdwgaLjabd6gaUjabdohaZjabdMgaPjabdsha0jabdMgaPjabdAha2jabdMgaPjabdsha0jabdMha5jabgwSixlabdcfaqjabdkfasbqaaiabdohaZjabdwgaLjabd6gaUjabdohaZjabdMgaPjabdsha0jabdMgaPjabdAha2jabdMgaPjabdsha0jabdMha5jabgwSixlabdcfaqjabdkfasjabgUcaRiabcIcaOiabigdaXiabgkHiTiabdohaZjabdchaWjabdwgaLjabdogaJjabdMgaPjabdAgaMjabdMgaPjabdogaJjabdMgaPjabdsha0jabdMha5jabcMcaPiabgwSixlabcIcaOiabigdaXiabgkHiTiabdcfaqjabdkfasjabcMcaPaaaaaa@7536@

and negative predictive value (NPV)

NPV=specificity⋅(1−PR)specificity⋅(1−PR)+(1−sensitivity)⋅PR
 MathType@MTEF@5@5@+=feaafiart1ev1aaatCvAUfKttLearuWrP9MDH5MBPbIqV92AaeXatLxBI9gBaebbnrfifHhDYfgasaacH8akY=wiFfYdH8Gipec8Eeeu0xXdbba9frFj0=OqFfea0dXdd9vqai=hGuQ8kuc9pgc9s8qqaq=dirpe0xb9q8qiLsFr0=vr0=vr0dc8meaabaqaciaacaGaaeqabaqabeGadaaakeaacqWGobGtcqWGqbaucqWGwbGvcqGH9aqpdaWcaaqaaiabdohaZjabdchaWjabdwgaLjabdogaJjabdMgaPjabdAgaMjabdMgaPjabdogaJjabdMgaPjabdsha0jabdMha5jabgwSixlabcIcaOiabigdaXiabgkHiTiabdcfaqjabdkfasjabcMcaPaqaaiabdohaZjabdchaWjabdwgaLjabdogaJjabdMgaPjabdAgaMjabdMgaPjabdogaJjabdMgaPjabdsha0jabdMha5jabgwSixlabcIcaOiabigdaXiabgkHiTiabdcfaqjabdkfasjabcMcaPiabgUcaRiabcIcaOiabigdaXiabgkHiTiabdohaZjabdwgaLjabd6gaUjabdohaZjabdMgaPjabdsha0jabdMgaPjabdAha2jabdMgaPjabdsha0jabdMha5jabcMcaPiabgwSixlabdcfaqjabdkfasbaaaaa@7863@

to assess the performance of our approach assuming a reasonable prevalence (PR). In addition, we computed receiver operator characteristic curves, plots of sensitivity versus 1-specificity. The value of interest is the area under the ROC curve, denoted as AUC value. For optimal classifications, AUC equals 1, for random classifications, AUC equals 0.5. The AUC serves as very meaningful performance measure.

## Authors' contributions

AK performed analyses and classification and helped to draft the manuscript. NL carried out the screening of the sera. NC participated in the design of the study and helped to write the manuscript. AH participated in the development of the subset selection method. EM and HPL designed the study and equally contributed as senior authors. All authors read and approved the final manuscript.

## References

[B1] Vicini F, Vargas C, Abner A, Kestin L, Horwitz E, Martinez A (2005). Limitations in the use of serum prostate specific antigen levels to monitor patients after treatment for prostate cancer. J Urol.

[B2] Sidransky D (2002). Emerging Molecular Markers of Cancer. Nat Rev Cancer.

[B3] Tibshirani R, Hastie T, Narasimhan B, Soltys S, Shi G, Koong A, Le Q (2004). Sample classification from protein mass spectrometry, by 'peak probability contrasts'. Bioinformatics.

[B4] Koomen J, Shih L, Coombes K, Li D, Xiao L, Fidler I, Abbruzzese J, Kobayashi R (2005). Plasma Protein Profiling for Diagnosis of Pancreatic Cancer Reveals the Presence of Host Response Proteins. Clin Cancer Res.

[B5] Wang X, Yu J, Sreekumar A, Varambally S, Shen R, Giacherio D, Mehra R, Montie J, Pienta K, Sanda M, Kantoff P, Rubin M, Wei J, Ghosh D, Chinnaiyan A (2005). Autoantibody signatures in prostate cancer. N Engl J Med.

[B6] Erkanli A, Taylor D, Dean D, Eksir F, Egger D, Geyer J, Nelson B, Stone B, Fritsche H, Roden R (2006). Application of Bayesian Modeling of Autologous Antibody Responses against Ovarian Tumor-Associated Antigens to Cancer Detection. Cancer res.

[B7] Comtesse N, Zippel A, Walle S, Monz D, Backes C, Fischer U, Mayer J, Ludwig N, Hildebrandt A, Keller A, Steudel W, Lenhof H, Meese E (2005). Complex humoral immune response against a benign tumor: Frequent antibody response against specific antigens as diagnostic targets. Proc Natl Acad Sci USA.

[B8] Lee S, Obata Y, Yoshida M, Stockert E, Williamson B, Jungbluth A, Chen Y, Old L, Scanlan M (2004). Immunomic analysis of human sarcoma. Proc Natl Acad Sci USA.

[B9] Scanlan M, Simpson A, Old L (2004). The cancer/testis genes: review, standardization, and commentary. Cancer Immun.

[B10] Schreiber H, Wu T, Nachman J, Kast W (2002). Immunodominance and tumor escape. Semin Cancer Biol.

[B11] Chatterjee M, Mohapatra S, Ionan A, Bawa G, Ali-Fehmi R, Wang X, Nowak J, Ye B, Nahhas F, Lu K, Witkin SS, Fishman D, Munkarah A, Morris R, Levin N, Shirley N, Tromp G, Abrams J, Draghici S, Tainsky MA (2006). Diagnostic markers of ovarian cancer by high-throughput antigen cloning and detection on arrays. Cancer Res.

[B12] Kleihues P, Louis D, Scheithauer B, Rorke L, Reifenberger G, Burger P, Cavenee W (2002). The WHO classification of tumors of the nervous system. J Neuropathol Exp Neurol.

[B13] Petricoin E, Ardekani A, Hitt B, Levine P, Fusaro V, Steinberg S, Mills G, Simone C, Fishman D, Kohn E, LA L (2002). Use of proteomic patterns in serum to identify ovarian cancer. The Lancet.

[B14] Spinney L (2006). Cancer: Caught in time. Aug.

[B15] Hastie T, Tibshirani R, Friedman J (2001). The Elements of Statistical Learning. Aug, Springer.

[B16] Shannon C (1984). A Mathematical Theory of Communication. The Bell System Technical Journal.

[B17] Wilcoxon F (1945). Individual comparisons by ranking methods. Biometrics Bulletin.

[B18] Mann H, Whitney D (1947). On a test of whether one of 2 random variables is stochastically larger than the other. Ann Mat Stat.

[B19] Shapiro S, Wilk M (1965). An analysis of variance test for normality. Biometrika.

